# Validity and reliability of speed tests used in soccer: A systematic review

**DOI:** 10.1371/journal.pone.0220982

**Published:** 2019-08-14

**Authors:** Stefan Altmann, Steffen Ringhof, Rainer Neumann, Alexander Woll, Michael C. Rumpf

**Affiliations:** 1 Department for Performance Analysis, Institute of Sports and Sports Science, Karlsruhe Institute of Technology, Karlsruhe, Germany; 2 Department of Sport and Sport Science, University of Freiburg, Freiburg, Germany; 3 Department for Social and Health Sciences in Sport, Institute of Sports and Sports Science, Karlsruhe Institute of Technology, Karlsruhe, Germany; 4 Sport Performance Research Institute New Zealand, Auckland University of Technology, Auckland, New Zealand; University of Belgrade, SERBIA

## Abstract

**Introduction:**

Speed is an important prerequisite in soccer. Therefore, a large number of tests have been developed aiming to investigate several speed skills relevant to soccer. This systematic review aimed to examine the validity and reliability of speed tests used in adult soccer players.

**Methods:**

A systematic search was performed according to the PRISMA guidelines. Studies were included if they investigated speed tests in adult soccer players and reported validity (construct and criterion) or reliability (intraday and interday) data. The tests were categorized into linear-sprint, repeated-sprint, change-of-direction sprint, agility, and tests incorporating combinations of these skills.

**Results:**

In total, 90 studies covering 167 tests were included. Linear-sprint (n = 67) and change-of-direction sprint (n = 60) were studied most often, followed by combinations of the aforementioned (n = 21) and repeated-sprint tests (n = 15). Agility tests were examined fewest (n = 4). Mainly based on construct validity studies, acceptable validity was reported for the majority of the tests in all categories, except for agility tests, where no validity study was identified. Regarding intraday and interday reliability, ICCs>0.75 and CVs<3.0% were evident for most of the tests in all categories. These results applied for total and average times. In contrast, measures representing fatigue such as percent decrement scores indicated inconsistent validity findings. Regarding reliability, ICCs were 0.11–0.49 and CVs were 16.8–51.0%.

**Conclusion:**

Except for agility tests, several tests for all categories with acceptable levels of validity and high levels of reliability for adult soccer players are available. Caution should be given when interpreting fatigue measures, e.g., percent decrement scores. Given the lack of accepted gold-standard tests for each category, researchers and practitioners may base their test selection on the broad database provided in this systematic review. Future research should pay attention to the criterion validity examining the relationship between test results and match parameters as well as to the development and evaluation of soccer-specific agility tests.

## Introduction

The game structure of soccer has dramatically changed over the last decades towards a more and more dynamic and faster playing style [1]. Compared to years past, modern soccer is denoted by shorter ball contact times, increased passing rates, higher player density, and faster transitions [1]. The changes in game structure also place modified demands on the players. These alterations not only affect technical and tactical aspects but particularly the players’ speed requirements. From a physical perspective, the players have to perform several accelerations and sprints at maximal speed with and without changes of direction throughout a match [2–4]. Moreover, players are forced to possess rapid information processing and to make fast and accurate decisions in order to be successful [1]. This indicates that speed in soccer encompasses both physical and perceptual-cognitive components [5].

As indicated above, speed is widely accepted to play a crucial role in soccer [6,7]. Therefore, speed testing has become a standard component of performance assessments [2,8]. For this purpose, a multitude of running-based tests has been developed aiming to examine several speed skills and have been implemented in research and practice [2,9]. More specifically, these speed tests can be categorized into linear sprinting, change-of-direction sprinting, repeated sprinting, agility, and combinations of these categories. In this context, linear sprinting relates to straight-line sprinting over various distances, including acceleration and maximum speed phases [10]. Moreover, change-of-direction sprinting comprises preplanned whole-body changes of directions as well as rapid movements and direction changes of the limbs [11,12]. Repeated sprinting refers to short-duration sprints (< 10 s) interspersed with brief phases of recovery (< 60 s) [13]. Finally, agility is considered an open skill and has been defined as a „rapid whole-body movement with change of velocity or direction in response to a stimulus”[11]. While linear sprinting, change-of-direction sprinting, and repeated sprinting mainly represent physically-driven speed skills, agility refers to both physical and perceptual-cognitive aspects of speed [5,13]. These skills share a relatively low common variance with limited training transfer between each other being evident. Hence, they can be considered as rather independent [12,14–22]. Therefore, a comprehensive examination of speed should address all test categories.

From a practical perspective, the feasibility, equipment needed, and economical aspects represent important factors whether or not to choose a test. From a scientific perspective, however, tests should possess appropriate levels of psychometric properties, including validity and reliability, in order to be used with confidence and to be able to draw meaningful conclusions from test results [23,24]. While recent reviews have been published focusing on tests of motor abilities such as endurance [25] and strength [26] with regards to soccer, no overview on the validity and reliability of tests addressing speed skills is available.

Therefore, the aim of this systematic review is to review the available literature on speed tests used in soccer with a focus on the tests’ validity and reliability. The results of this review could help both scientists and practitioners decide which test(s) to choose depending on the specific aspects of speed being of interest.

## Methods

This systematic review was written according to the Preferred Reporting Items for Systematic Reviews and Meta-Analyses (PRISMA) guidelines [27]. The protocol was not registered prior to the initiation of the project.

### Literature search

A systematic review of the published literature was undertaken using the electronic databases PubMed and Web of Science during April and May 2018. An updated search regarding studies published after May 2018 was not conducted. The literature search was conducted by one researcher (SA). There was no restriction on publication date.

The following keywords were used to capture psychometric properties: psychometric, measurement.

The following keywords were used to capture validity: validity, logical, construct, convergent, discrimination, match performance, gold standard, level, standard.

The following keywords were used to capture reliability: reliability, repeatability, reproducibility, measurement error, consistency, smallest worthwhile change, minimal detectable change, typical error, usefulness.

The following keywords were used to capture speed testing and the different test categories: speed, quickness, sprint, acceleration, maximum speed, linear, change of direction, repeated sprint ability, agility, reactive agility, physical, unplanned, unanticipated, test, testing.

The following keywords were used to capture soccer: soccer, football.

Reference lists of retrieved full-text articles and recent reviews were examined to identify additional articles not identified by the initial search.

Eligibility criteria for study inclusion consisted of one of the following: (i) tests performed two or more times during one occasion (intraday reliability) or on two or more separate occasions (interday reliability); (ii) compared against other standards of play (construct validity); (iii) compared against match performance (criterion validity).

Except for reviews, all types of studies relating to at least one speed-test category (linear sprinting, repeated sprinting, change-of-direction sprinting, agility, and combinations) were taken into consideration. In addition, studies must have been published in English language in a peer-reviewed journal. As the present review focuses on adult players, only populations with a mean age of 17 years or older were considered. There was no restriction on gender (female and male) and playing level (e.g., recreational, amateur, semi-professional, professional). Complex tests incorporating passing or shooting were only considered when the part relating to speed was examined separately from the total test time. Studies investigating the factorial or convergent validity of speed tests were not included.

### Literature selection

The literature selection consisted of two screening phases. In phase one, duplicates, titles, and abstracts were screened. In phase two, the full papers were screened using the eligibility (inclusion) criteria noted above.

### Data extraction and analyses

Data were extracted independently by four researchers (SA, SR, RN, and MR) and documented using a Microsoft Excel 2016 spreadsheet (Microsoft Corporation, Redmond, Washington, USA). Extracted data from each study included publication details, number of participants, demographic information (including gender, age, playing level, and country), test category, test name, short test description, type, outcome measures as well as results for validity or reliability, respectively, and the information required to assess the methodological quality of each study. If more than one group of players were investigated in a study, only the groups with a mean age of 17 years or older were considered.

For reliability (both intraday and interday), intraclass correlation coefficient (ICC), Pearson’s r, and coefficient of variation (CV) values were recorded. While ICC and Pearson’s r represent relative reliability, CV is a measure of absolute reliability. By reflecting the degree to which indivuduals in a specific sample maintain their position over the course of repeated trials (interindividual variability), measures of relative reliability are affected by group homogeneity. Conversely, measures of absolute reliability relate to the variation over repeated trials within individuals (intraindividual variability). Therefore, they do not depend on group homogeneity [28]. Considering the ICC, a range of different approaches exist on how to interpret these values [28]. Following the recommendations of a review with a similar objective [29], in the present review, “good” reliability was considered ICC ≥ 0.75. This value was chosen as it appears to reflect a reasonable consensus as to what can be considered good reliability. The same value was applied for Pearson’s r. While a threshold of 10% for acceptable CV values has been suggested, this number seems rather arbitrary [28]. Therefore, CV values were interpreted in relation to each other.

Relating to construct validity, where possible, the percentage difference between playing levels and the respective effect sizes (ES) were calculated and rated according to Hopkins [30]. An ES less than 0.2 was considered a trivial effect; 0.2 ≤ ES < 0.6 a small effect; 0.6 ≤ ES < 1.2 a moderate effect; 1.2 ≤ ES < 2.0 a large effect; 2.0 ≤ ES < 4.0 a very large effect; and ≥ 4.0 an extremely large effect. In terms of criterion validity, the magnitude of the correlation coefficient between speed-test results and match parameters was considered as small (0.1 ≤ r < 0.3), moderate (0.3 ≤ r < 0.5), large (0.5 ≤ r < 0.7), very large (0.7 ≤ r < 0.9), and nearly perfect (r ≥ 0.9) [30].

Data were checked and verified by SA and discrepancies were resolved through discussion. The synthesis of the results was carried out descriptively.

### Assessment of methodological quality

The methodological quality of the studies included in the review was assessed through a modified version of the critical appraisal tool [31]. The modified checklist included nine items:

1. Subject characteristics were clearly described (validity and reliability studies)
2. Competence of the raters was clearly described (validity and reliability studies)
3. Reference (match data) was clearly described (criterion validity studies)
4. Raters were blinded to their own prior findings (reliability studies)
5. Time interval between the reference (match data) was suitable (criterion validity studies)
6. Time interval between repeated measures was suitable (reliability studies)
7. Test execution was described in sufficient detail to permit replication of the test (validity and reliability studies)
8. Methodological aspects (e.g., timing technology, starting position, surface) were described in sufficient detail to permit replication of the test (validity and reliability studies)
9. Statistical methods were appropriate for the purpose of the study (validity and reliability studies)

From the original checklist, the items 6 (Variation of order of examination), 9 (Independence of reference standard from index test), and 12 (Explanation of withdrawals) were not included as they were thought to be not appropriate for the purpose of this review. Conversely, item 8 (Methodological aspects) was added to the checklist because of the considerable influence of methodological aspects on results, validity, and reliability of speed tests [32]. Due to the large absolute errors associated with manual timing through stopwatches, tests using this timing technology were excluded [32].

The score for each item was determined as follows: 2 = clearly yes; 1 = to some extent; 0 = clearly no. Consequently, the maximal possible score was 14 (criterion) and 10 (construct) for validity studies, and 14 (intraday and interday) for reliability studies. In the case of more than one test being examined in a single study, the score was calculated for each test separately. According to Barrett et al. [33], the methodological quality was rated as high when > 60% of the maximal possible score was obtained (corresponding to a score of > 6 for construct validity studies and > 8.4 for criterion validity, intraday, and interday reliability studies).

## Results

### Search results

**Fig 1:**
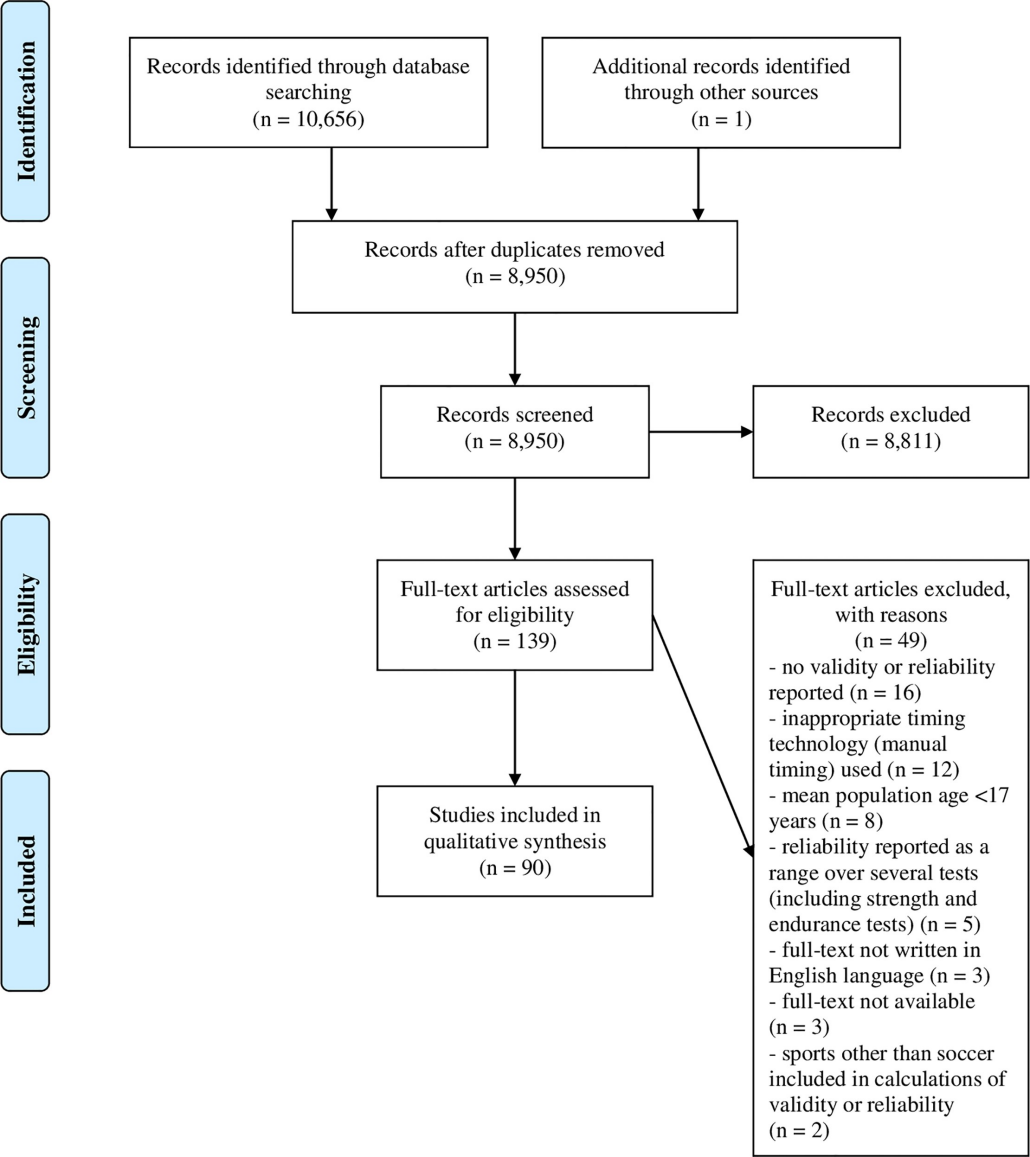
Flow diagram of the search and selection process for inclusion of articles.

A flow diagram for the selection of the studies can be found in Fig 1. 10,656 records were retrieved through the initial search in the electronic databases. The removing of duplicates yielded 8,950 studies that were screened for the title. Subsequent abstract screening (1,270 records) led to the exclusion of further 1,131 studies. Consequently, the full-texts of 139 articles were assessed for eligibility, with 49 articles being excluded. The reasons for exclusion during full-text screening were

■ no validity or reliability reported (16 studies),
■ inappropriate timing technology (manual timing) used (12 studies),
■ mean population age < 17 years (8 studies),
■ reliability reported as a range over several tests (including strength and endurance tests) (5 studies),
■ full-text not written in English language (3 studies),
■ full-text not available (3 studies), and
■ sports other than soccer included in calculations of validity or reliability (2 studies).

Ultimately, 90 studies were included in this review.

### Overview on studies and tests included

From the 90 studies included, 20 referred to validity only, 60 to reliability only, and 10 to both validity and reliability. An overview on the number of the tests regarding validity and reliability in each category is presented in Table 1. Ball dribbling was included in change-of-direction sprint tests (4 validity, 3 reliability) and in combinations (1 validity). A total of 3,901 participants (mean ± standard deviation 56 ± 108, median 25, range 7–939) with an average age from 17 to 33 years (mean ± standard deviation 21 ± 3 years, median 21 years) were involved. Most studies examined male players (74), while female (13) and both male and female players (3) were studied less often. The playing level covered a wide range between recreational and national team players.

**Table 1:** Overview.

	Number of tests	
Test category	Validity (Construct/Criterion)	Reliability (Intraday/Interday)
Linear sprint	16 (14/2)	51 (26/22)*
Repeated sprint	6 (5/1)	9 (2/6)*
Change of direction	15 (14/1)	45 (27/16)*
Agility	0 (0/0)	4 (0/4)
Combinations	10 (8/2)	11 (2/8)*
**Total**	**47 (41/6)**	120 (57/57)*

*–Deviating sum due to reliability type being not specified for each test.

### Assessment of methodological quality

Construct and criterion validity were reported for 41 and 6 tests, respectively. The mean score was 6.4/10 (range 4–10) and 9.8/14 (range 9–12) leading to a high rating of methodological quality.

Intraday and interday reliability were reported for 57 and 56 tests, respectively, with reliability type being not specified for 7 tests. The mean score was 7.9/14 (range 5–11) and 7.8/14 (range 5–11), which is below the threshold for a high rating of methodological quality (Tables 2–6, column ‘MQ’).

**Table 2:** Linear-sprint tests (validity).

Study	Population				Short description	Type	Results	MQ
	N	Gender	Age	Playing level (Country)				
Silva et al. [34]	13	male	25.7 ± 4.6	Portuguese championship (Portugal)	30-m sprint, split at 5 m; high-intensity running and sprinting during matches	Criterion	5 m and high-intensity running during matches: r = -0.40 –-0.67 5 m and sprinting during matches: r = -0.56 –-0.62 30 m and high-intensity running during matches: r = -0.35 –-0.63 30 m and sprinting during matches: r = -0.46 –-0.73	10 (14)
Djaoui et al. [35]	48	male	Professional: 24.3 ± 2.6 Elite amateur: 20.9 ± 2.9	Professional, elite amateur (France)	40-m sprint, with GPS; maximal sprinting speed during matches	Criterion	Maximal sprinting speed during 40-m sprint and matches: r = 0.52	9 (10)
					40-m sprint, with GPS	Construct	Elite amateur faster than professional (1.6%, ES (d) = 0.33)	6 (10)
Haugen et al. [36]	939	male	22.1 ± 4.3	National team, 1st division, 2nd division, 3rd to 5th division, junior national team, juniors (Norway)	40-m sprint, splits at 10, 20, and 30 m	Construct	20 m: National team faster than 2nd division (1.4%, ES (d) = 0.50), 3rd to 5th division (3.8%, ES (d) = 1.20), junior national team (1.8%, ES (d) = 0.60), and junior players (2.8%, ES (d) = 0.90) Fastest 10-m split: "similar results"	8 (10)
Ferro et al. [37]	42	male	21.2 ± 1.70	Competitive, non-competitive (not specified)	30-m sprint, 10-m sections, with laser system	Construct	Competitive always better than non-competitive* 10 m: 0.7%, ES = 0.23 10–20 m: 1.4%, ES = 0.42 20–30 m: 1.8%, ES = 0.42 20 m: 1.1%, ES = 0.36 30 m: 1.3%, ES = 0.39 10–30 m: 1.6%, ES = 0.44	6 (10)
Silvestre et al. [38]	25	male	19.9 ± 1.3	Starters and non-starters of a division 1 team (USA)	36.5-m sprint, split at 9.1 m	Construct	Pre-Season 36.5 m: Starters better than non-starters (2%, ES (d) = 0.52) Pre-Season 9.1 m and Post-Season 9.1 m and 36.5 m: No differences between groups	5 (10)
Silvestre et al. [39]	27	male	19.9 ± 1.3	Starters and non-starters of a division 1 team (USA)	36.5-m sprint, split at 9.1 m	Construct	36.5 m: Starters better than non-starters (3.9%, ES (d) = 1.04) 9.1 m: No differences between groups	5 (10)
Cometti et al. [40]	95	male	1st division: 26.1 ± 4.3 2nd division: 23.2 ± 5.6 amateurs: 25.8 ± 3.9	1st division, 2nd division, amateurs of regional standard (France)	30-m sprint, split at 10 m, visual stimulus as a starting signal	Construct	10 m: 1st division faster than 2nd division (0.8%, ES (d) = 0.24) and amateur (3.0%, ES (d) = 0.8) 30 m: 1st division faster than 2nd division (0.6%, ES (d) = 0.16) and amateur (1.7%, ES (d) = 0.43)	6 (10)
Risso et al. [41]	22	female	Starters: 20.4 ± 1.3 Non-starters: 20.1 ± 1.2	Starters and non-starters of professional team (USA)	30-m sprint, splits at 5 m and 10 m	Construct	Starters always better than non-starters 5 m: 0.9%, ES (d) = 0.2 10 m: 0.5%, ES (d) = 0.16 30 m: 2.1%, ES (d) = 0.64	6 (10)
Vescovi [42]	140	female	23.9 ± 2.8	Drafted and non-drafted players of try-outs of a professional women's soccer league (USA)	35-m sprint, splits at 5 m, 10 m, and 20 m	Construct	Drafted always better than non-drafted* 5 m: 4.1%; ES (d) = 0.55 10 m: 2.8%; ES (d) = 0.56 20 m: 2.9%; ES (d) = 0.67 35 m: 3%; ES (d) = 0.78	10 (10)
Rebelo et al. [43]	180	male	18.2 ± 0.6	1st division elite, regional division non-elite (Portugal)	30-m sprint, split at 5 m	Construct	Elite better than non-elite** 5 m: 4.6%; ES (d) = 0.57 30 m: 1.48%; ES (d) = 0.38	7 (10)
Haugen et al. [44]	194	female	22 ± 4.1	Senior national-team, 1st division, 2nd division, highest junior division (Norway)	40-m sprint, splits at 10, 20, and 30 m	Construct	20 m: National team faster than 1st division (2%, ES (d) = 0.5) and 2nd division (5%, ES (d) = 1.30); 1st division faster than 2nd division (3%, ES (d) = 0.80); junior elite faster than 2nd division (3%, ES (d) = 0.80) 20–40 m: National team faster than 2nd division (5.0%, ES (d) = 1.10); 1st division faster than 2nd division (3.0%, ES (d) = 0.70)	8 (10)
Cotte & Chatard [45]	14	male	International: 24.2 ± 6.1 National: 26.5 ± 5.9	International and national players of English premier league team (England)	30-m sprint, splits at 10 and 20 m	Construct	International always better than national, except for velocity 10–20 m Time: 10 m (1.2%, ES (d) = 0.25), 20 m (1.0%, ES (d) = 0.3), 30 m (1.2%, ES (d) = 0.32) Velocity: 0–10 m (1.2%, ES (d) = 0.26), 10–20 m (0.1%, ES (d) = 0.04), 20–30 m (1.2%, ES (d) = 0.29)	7 (10)
Nikolaidis et al. [46]	179	male	U18–U35: 17.48 ± 0.23–32.58 ± 1.77	2nd, 3rd, and 4th national leagues (Greece)	20-m sprint	Construct	U19 better than U20 (0.6%), U25 (0.6%), U35 (2.8%), U21 (3.4%), U30 (3.7%), and U18 (4.0%)	7 (10)
Mujika et al. [47]	68	male, female	Female: 17 ± 1.6 (junior) 23.1 ± 2.9 (senior) Male: 18.4 ± 0.9 (junior), 24 ± 3.4 (senior)	Senior females of Spanish Super Liga, junior females of Primera Nacional, senior males of La Liga, junior of Tercera Division (Spain)	15-m sprint	Construct	Female: Senior better than junior (2.1%, ES (d) = 0.64) Male: Junior better than senior (0.1%, ES (d) = 0.05)	7 (10)
Kobal et al. [48]	45	male	Professional: 22 ± 2.9 U20: 19 ± 0.6	Professional, U20 (Brazil)	20-m sprint, split at 10 m	Construct	10 m: U20 better than professional (ES (d) = 0.14) 20 m: Professional better than U20 (ES (d) = 0.38)	7 (10)

MQ–Methodological quality, maximal possible score in parenthesis; ES–Effect size; GPS–Global positioning system

*–Selected parameters

**–Pooled ES for several positions

**Table 3:** Linear-sprint tests (reliability).

Study	Population				Short description	Type	Results	MQ
	N	Gender	Age	Playing level (Country)				
Gelen [49]	26	male	23.2 ± 3.2	Professionals from 3rd division (Turkey)	30-m sprint	Intraday	ICC = 0.87–0.91	7 (14)
Rouissi et al. [50]	31	male	17.42 ± 0.55	Professionals from 1st division (Tunisia)	10-m sprint	Interday	ICC = 0.94; CV = 1.6%	9 (14)
Haugen et al. [51]	30 (m), 14 (f)	male, female	18.2 ± 1.0	Amateurs	25-m and 40-m sprint Flying start distances: 0.5, 1, 1.5, 2, 5, 10, 15 m	Intraday	20-m sprint time with flying start: 0.5 m: ICC = 0.99; CV = 1.2% 1 m: ICC = 0.99; CV = 1.3% 1.5 m: ICC = 0.99; CV = 1.3% 2 m: ICC = 0.99; CV = 1.4% 5 m: ICC = 0.99; CV = 1.0% 10 m: ICC = 0.99; CV = 1.0% 15 m: ICC > 0.99; CV = 0.9% 10-m sprint time with flying start: Similar trend but with slightly higher CV values across all flying-start distances (1.4–1.8%)	10 (14)
López-Segovia et al. [52]	21	male	18.4 ± 0.8	Professional from Spanish national league division (Spain)	30-m sprint	Interday	ICC = 0.90; CV = 1.1%	8 (14)
Pareja-Blanco et al. [53]	21	male	24.3 ± 4.6	Professional Moroccan soccer club (Morocco)	30-m sprint	Intraday	ICC = 0.98; CV = 0.8%	8 (14)
Sporis et al. [54]	270	male	28.3 ± 5.9	Professionals from 1st national league (Croatia)	20-m sprint, splits at 5 and 10 m	Intraday	5 m: ICC = 0.89 10 m: ICC = 0.80 20 m: ICC = 0.81	5 (14)
Zois et al. [55]	10	male	23.3 ± 2.5	Amateurs from Serie D (Italy)	20-m sprint	Interday	CV = 0.8%	8 (14)
Emmonds et al. [56]	10	female	25.4 ± 7.0	Professional from highest division (WSL1) (England)	30-m sprint, splits at 10 and 20 m	Intraday	10 m: ICC = 0.95; CV = 1.4% 20 m: ICC = 0.92; CV = 1.3% 30 m: ICC = 0.90; CV = 1.5%	8 (14)
Mujika et al. [57]	20	male	18.3 ± 0.6	Professional juniors at national level (not specified)	15-m sprint	Intraday	ICC = 0.94	9 (14)
Loturco et al. [58] & Loturco et al. [59]	27	male	18.4 ± 1.2	Professional U20, São Paulo state elite championship (Brazil)	30-m sprint	Intraday	ICC = 0.97; CV = 2.3%	8 (14) 9 (14)
Boone et al. [60]	289	male	25.4 ± 4.9	Professionals from 1st division (Belgium)	10-m sprint, split at 5 m; auditory cue as a starting signal	Intraday	5 m: ICC = 0.88 10 m: ICC = 0.90	5 (14)
Manson et al. [61]	33	female	U19: 17.8 ± 0.71 Senior: 23.3 ± 4.89	Professionals from national team (New Zealand)	Linear sprint for > 6 s on a nonmotorized treadmill	Intraday	Velocity: ICC = 0.79; CV = 2.0%	7 (14)
Meylan et al. [62]	20	female	18.2 ± 0.7	Professionals from national team (Top 10 in the world)	40-m sprint, with timing lights and GPS	Intraday	Timing lights: ICC = 0.80–0.96; CV = 0.9–2.3% GPS: ICC = 0.86; CV = 2.1%	6 (14)
Sjökvist et al. [63]	14	female	20.3 ± 2.3	Collegiate players from NCAA division 1 (USA)	20-m sprint	Interday	ICC > 0.93	8 (14)
Requena et al. [64]	14	male	20.0 ± 3.6	Professional (not specified)	15-m sprint	Interday	ICC = 0.87–0.95	7 (14)
Ingebrigtsen et al. [65]	57	male	22 ± 5	Professionals from 3 best leagues (Norway)	35-m sprint, splits at 10 and 20 m	Intraday	10 m: ICC = 0.94; CV = 0.7% 20 m: ICC = 0.97; CV = 1.4% 35 m: ICC = 0.96; CV = 1.9%	8 (14)
Yanci et al. [66]	39	male	22.9 ± 2.8	Professionals from 3rd division (Spain)	15-m sprint, splits at 5 and 10 m	Intraday	5 m: CV = 2.5% 10 m: CV = 1.7% 15 m: CV = 1.2%	9 (14)
Comfort et al. [67]	34	male	17.2 ± 0.6	Well-trained players (England)	20-m sprint, split at 5 m	Intraday	5 m: ICC = 0.87 20 m: ICC = 0.97	7 (14)
López-Segovia et al. [68]	14	male	20.14 ± 0.4	Amateur (not specified)	30-m sprint, splits at 10 and 20 m	Intraday	10 m, 20 m, 30 m, 10–20 m, 10–30 m, 20–30 m: ICC: 0.92–0.99; CV: 1.2–2.6%	7 (14)
Chelly et al. [69]	23	male	17.2 ± 0.7	Semi-professionals from national junior championship league (Tunesia)	10-m sprint, with camera at 25 frames per second	Intraday	Sprint velocities and accelerations: ICC = 0.87–0.96	8 (14)
Spierer et al. [70]	15	male	22.1 ± 1.5	Professionals from Division 1 (USA)	20-m linear sprint with auditory stimulus as a starting signal	Intraday	ICC = 0.97	7 (14)
Caldwell & Peters [71]	13	male	24 ± 4.4	Semi-professionals from nationwide conference north league (England)	15-m sprint	Interday	ICC = 0.80	7 (14)
Ronnestad et al. [72]	21	male	IG1: 23 ± 2 IG2: 22 ± 2.5 CG: 24 ± 1.5	Professional (Norway)	40-m sprint, splits at 10 and 30 m	Intraday	10 m and 30–40 m: CV < 3.0%	7 (14)
Small et al. [73]	9	male	21.3 ± 2.9	Semi-professional (United Kingdom)	10-m sprint, test is part of a soccer match simulation	Intraday	ICC > 0.83	6 (14)
Los Arcos et al. [21]	42	male	23.2 ± 2.4	Professionals from 2nd and 3rd division (Spain)	15-m sprint, splits at 5 and 10 m	Intraday	5 m: ICC = 0.87 10 m: ICC = 0.93 15 m: ICC = 0.96	9 (14)
Gorostiaga et al. [74]	19	male	17.2; range: 16–18.5	Amtaeurs at regional level (Spain)	15-m sprint	Intraday	CV < 1.5%	9 (14)
Gil et al. [75]	20	male	23.3 ± 4.8	Professional (Brazil)	25-m sprint	Interday	ICC = 0.92; CV = 1.3%	6 (14)
Coelho et al. [76]	138	male	U17: 17.3 ± 5.33 U20: 20.6 ± 3.66 Professional: 23.25 ± 6.42	Professionals from 1st division (Brazil)	30-m sprint, splits at 10 and 20 m	Interday	10 m: ICC = 0.98 20 m: ICC = 0.96 30 m: ICC = 0.96	9 (14)
Boussettaa et al. [77]	11	male	21.82 ± 0.51	Healthy players (not specified)	10-m sprint	Interday	ICC = 0.88	7 (14)
Shalfawi et al. [78]	20	female	19.4 ± 4.4	Well-trained players from 2nd division (Norway)	40-m sprint	Intraday	ICC = 0.83	8 (14)
Sayers et al. [79]	20	female	19.35 ± 0.99	Professional from women‘s professional socer league (not specified)	30-m sprint, splits at 10 and 20 m	Interday	30 m: ICC > 0.99 20–30 m: ICC = 0.99 10 m: ICC = 0.99	9 (14)
Thomas et al. [80]	12	male	17.3 ± 0.4	Semi-professionals from soccer academy (United Kingdom)	20-m sprint	Interday	5 m: ICC = 0.93 10 m: ICC = 0.96 15 m: ICC = 0.94 20 m: ICC = 0.98	8 (14)
Iaia et al. [81]	18	male	18.5 ± 1.0	Professionals at national level (Denmark)	200-m sprint	Interday	CV = 0.8%	8 (14)
Rey et al. [82]	18	male	26.6 ± 3.7	Professional (Spain)	10-m sprint, split at 5 m	Interday	5 m: ICC = 0.96 10 m: ICC = 0.94	7 (14)
McGawley & Andersson [83]	18	male	23.0 ± 4.0	Semi- and fully-professional from 1st division (Sweden)	10-m sprint	Interday	CV = 1.8%	7 (14)
Loturco et al. [84]	24	male	18.2 ± 0.6 and 18.5 ± 0.8	High-level U20 players (Brazil)	20-m sprint, split at 10 m	Interday	10 m, 20 m, 10–20 m: ICC > 0.98	7 (14)
Rebelo et al. [43]	180	male	18.2 ± 0.6	1st division elite, regional division non-elite (Portugal)	30-m sprint, split at 5 m	Intraday	5 m: ICC = 0.97 30 m: ICC = 0.97	6 (14)
Silva et al. [34]	13	male	25.7 ± 4.6	Professional Portuguese championship team (Portugal)	30-m sprint, split at 5 m	Not specified	5 m and 10 m: ICC: 0.76–0.87	6 (14)
Kobal et al. [48]	45	male	Professional: 22 ± 2.9 U20: 19 ± 0.6	Professional, U20 (Brazil)	20-m sprint	Intraday	ICC = 0.88	7 (14)
Silva et al. [85]	18	male	25.7 ± 4.6	Professionals from Portuguese elite championship (Portugal)	30-m sprint	Not specified	ICC = 0.71–0.87	6 (14)
Bullock et al. [86]	18	male	18 ± 3	High-level amateurs from local soccer clubs (not specified)	5-m sprint; test is a part of a complex test	Interday	ICC = 0.55; r = 0.60; CV = 2.9%	8 (14)
Cotte & Chatard [45]	14	male	International: 24.2 ± 6.1 National: 26.5 ± 5.9	International and national players of English premier league team (England)	30-m sprint, splits at 10 and 20 m	Intraday	10 m: CV = 5.2% 20 m: CV = 4.8% 30 m: CV = 4.5%	9 (14)
Williams et al. [87]	15	male	26.1 ± 4.6	Amateurs from local league (not specified)	20-m sprint, split at 12 m, test is part of a soccer match simulation	Interday	12 m: CV = 1.8–3.2% 20 m: CV = 0.9–3.3%	8 (14)
Mirkov et al. [88]	20	male	20.4 ± 1.8	Professionals from 1st selection of a premier national league team (Serbia)	30-m sprint, split at 10 m	Intraday	10 m: ICC = 0.81; CV = 3.2% 10–30 m: ICC = 0.93; CV = 2.1%	9 (14)
Silva et al. [89]	7	male	22–31	Professionals from Portuguese soccer league (Portugal)	30-m sprint	Intraday	CV = 3.5%	7 (14)
Kutlu et al. [90]	34	female	20.8 ± 1.9	Amateurs from university soccer team (Turkey)	20-m sprint	Interday	ICC = 0.94; CV = 4.0%	9 (14)
Harper et al. [91]	10	male	22 ± 3	University-standard (England)	20-m sprint, test is part of a soccer match simulation	Interday	CV = 0.5–3.5%	7 (14)
Sonderegger et al. [92]	72	male	17.1 ± 0.6	Highly-trained U18 top level teams (Switzerland)	50-m sprint, velocity at the start line: 0 km/h, 6 km/h, 10.8 km/h, and 15 km/h	Interday	0 km/h: CV = 6.7% 6 km/h: CV = 5.4% 10.8 km/h: CV = 6.1% 15 km/h: CV = 10.9%	9 (14)
Ispirlidis et al. [93]	24	male	21.1 ± 1.2	Elite (Greece)	20-m sprint	Not specified	CV = 3.5%	5 (14)
Yanci et al. [94]	12	male	21.08 ± 1.57	Amateur (Spain)	20-m sprint, with timing lights and GPS	Interday	Timing lights: ICC = 0.73; CV = 1.9% GPS: ICC = 0.17; CV = 7.8%	9 (14)
Haugen et al. [44]	194	female	22 ± 4.1	Senior national-team, 1st division, 2nd division, highest junior division (Norway)	40-m sprint, splits at 10, 20, and 30 m	Interday	Long-term reliability (6–12 months) 10 m: r = 0.77; CV = 2.9% 10–20 m: r = 0.82; CV = 2.6% 20–30 m: r = 0.85; CV = 3.3% 30–40 m: r = 0.81; CV = 1.8% 20 m: r = 0.90; CV = 2.1% 20–40 m: r = 0.80; CV = 3.3%	9 (14)

MQ–Methodological quality, maximal possible score in parenthesis; ICC–Intraclass correlation coefficient, r–Pearson’s r; CV–Coefficient of variation; IG–Intervention group; CG–Control group; GPS–Global positioning system

**Table 4:** Repeated-sprint tests (validity).

Study	Population				Short description	Type	Results	MQ
	N	Gender	Age	Playing level (Country)				
Carling et al. [95]	12	male	Senior (not specified	1st French league (France)	6 x 6-s sprints, 20 s passive recovery, on a non-motorized treadmill	Criterion	Test: AV, HAV, PV, %Dec Match: High-intensity actions (% of total distance covered, average number, average recovery time, % of recovery time < 20 s and < 30 s), repeated high-intensity bouts (average number, average velocity, maximum velocity) %Dec and % of high-intensity actions with recovery times ≤ 20 s: r = -0.51 Average velocity and recovery time between high-intensity actions: r = 0.42 No further notable correlations between repeated-sprint test parameters and match parameters*	9 (14)
Dellal & Wong [96]	26	male	Senior, U19 (not specified)	2nd senior team of a professional club, U19 (France)	10 x 20-m sprints, 25 s active recovery	Construct	2nd team always better than U19 FT: 2.7%; ES (d) = 0.90 AT: 2.6%; ES (d) = 0.88 TT: 2.6%; ES (d) = 0.89 %Dec: 6.0%; ES (d) = 0.15	5 (10)
Wong et al. [97]	34	male	23.3 ± 3.6	Professional, college (not specified)	6 × 20-m sprints, 25 s active recovery	Construct	Professional always better than college: FT: 2.2%; ES (d) = 0.69 AT: 2.4%; ES (d) = 0.77 TT: 2.6%; ES (d) = 0.84 %Dec: 14.5%; ES (d) = 0.39	5 (10)
Gabbett [98]	19	female	18.1 ± 2.9	Professional, semi-professional (Australia)	Game-specific test of repeated-sprint ability for elite women's soccer players: 6 × 20-m sprints, starting every 15 s, 20 m active recovery	Construct	Professional always better than semi-professional: IT: 8.3%; ES (d) = 3.17 TT: 10.3%; ES (d) = 5.50 %Dec: 8.8%; ES (d) = 0.30	5 (10)
Aziz et al. [99]	52	male	Professional: 20.4 ± 1.6 Amateur: 23.5 ± 0.8	Professional U23 players, amateur players from a university (Singapore)	6 × 20-m sprints, 20 m active recovery	Construct	Professional always better than amateur: FT: 0.3%, ES (d) = 0.14 TT: 2.3%, ES (d) = 0.83	7 (10)
Ingebrigtsen et al. [100]	51	male	Elite: 26 ± 7 Sub-elite: 20 ± 3	Elite 1st division players, Sub-elite 3rd division players (Norway)	7 x 35-m sprints, 25 s active recovery	Construct	AT: Elite better than sub-elite (0.4%; ES (d) = 0.1) %Dec: Sub-elite better than elite: (22.9%; ES (d) = 0.4)	4 (10)

MQ–Methodological quality, maximal possible score in parenthesis; ES–Effect size

*–Selected parameters

AV–Average velocity; HAV–Highest average velocity; PV–Peak velocity; %Dec–Percent decrement; FT–Fastest time; AT–Average time; TT–Total time; IT–Initial time

**Table 5:** Repeated-sprint tests (reliability).

Study	Population				Short description	Type	Results	MQ
	N	Gender	Age	Playing level (Country)				
Chaouachi et al. [101]	23	male	19 ± 1	Professionals from national leagues (Tunisia)	7 x 30-m sprints, 25 s recovery	Not specified	TT: ICC = 0.92; CV = 2.7%	9 (14)
Haugen et al. [102]	25	male female	17 ± 1	1st junior division high school (Norway)	12 x 20-m sprints, starting every 60 s	Intraday	TT: CV = 0.8%	5 (14)
Shalfawi et al. [78]	20	female	19.4 ± 4.4	Well-trained from 2nd division (Norway)	7 x 30-m sprint, 30 s recovery	Interday	TT: ICC = 0.91	8 (14)
Iaia et al. [81]	18	male	18.5 ± 1.0	Professionals at national level (Denmark)	15 x 40 m-sprint, 30 s passive recovery	Interday	TT: CV = 1.2% %Dec: CV = 16.8%	6 (14)
McGawley & Andersson [83]	18	male	23.0 ± 4.0	Semi- and fully-professional from 1st division (Sweden)	6 x 30 m-sprints, starting every 20 s	Interday	TT: CV = 0.8%	7 (14)
López-Segovia et al. [103]	19	male	21.2 ± 2.1	Semi-professional from 3rd division (Spain)	Repeated 40-m sprints, splits at 10 and 20 m, until there was a 3% decrease in performance, 2 min passive recovery	Interday	TT: 10 m, 20 m, and 40 m: ICC = 0.92–0.99; CV = 1.2–2.6%	7 (14)
Wong et al. [97]	34	male	23.3 ± 3.6	Professional, college (not specified)	6 × 20-m sprints, 25 s active recovery	Interday	FT: ICC = 0.88; CV = 5.0% AT: ICC = 0.90; CV = 5.0% TT: ICC = 0.90; CV = 5.0% %Dec: ICC = 0.11; CV = 46.0%	7 (14)
Gabbett [98]	19	female	18.1 ± 2.9	Professional, semi-professional (Australia)	Game-specific test of repeated-sprint ability for elite women's soccer players: 6 × 20-m sprints, starting every 15 s, 20 m active recovery	Interday	TT: ICC = 0.91; CV = 1.5% %Dec: ICC = 0.14; CV = 19.5%	7 (14)
Ruscello et al. [104]	17	male	21.9 ± 3.6	Professionals from Italian lega pro (Italy)	7 x 30-m sprints, exercise to rest ratio 1:5, passive recovery	Intraday	TT: ICC = 0.75	6 (14)

MQ–Methodological quality, maximal possible score in parenthesis; ES–Effect size

*–Selected parameters

%Dec–Percent decrement; FT–Fastest time; AT–Average time; TT–Total time, ICC–Intraclass correlation coefficient, r–Pearson’s r; CV–Coefficient of variation

**Table 6:** Change-of-direction sprint tests (validity).

Study	Population				Short description	Type	Results	MQ
	N	Gender	Age	Playing level (Country)				
Silva et al. [34]	13	male	25.7 ± 4.6	Professional Portuguese championship team (Portugal)	T Test (36.6 m): Linear sprinting (9.1 m), COD of 90° to the left (4.6 m), COD of 180° (9.1 m), COD of 180° to the left (4.6 m), COD of 270° to the left (9.1 m)	Criterion	T Test and high-intensity running during matches: r = -0.01 –-0.56 T Test and sprinting during matches: r = -0.15 –-0.34	10 (14)
Mujika et al. [47]	68	male, female	Female: 17 ± 1.6 (junior) 23.1 ± 2.9 (senior) Male: 18.4 ± 0.9 (junior), 24 ± 3.4 (senior)	Senior females of Spanish Super Liga, junior females of Primera Nacional, senior males of La Liga, junior of Tercera Division (Spain)	15-m sprint: Linear sprinting (3 m), slalom section (3 m), clearing a hurdle (2 m), linear sprinting (7 m) to the finish	Construct	Male: Senior better than junior (5.1%, ES (d) = 1.27) Female: Senior better than junior (7.8%, ES (d) = 1.47)	7 (10)
					15-m ball dribbling: 15-m sprint, while dribbling and kicking a ball	Construct	Male: Senior better than junior (0.8%, ES (d) = 0.11) Female: Senior better than junior (12.2%, ES (d) = 1.41)	7 (10)
Risso et al. [41]	22	female	Starters: 20.4 ± 1.3 Non-starters: 20.1 ± 1.2	Starters and non-starters of professional team (USA)	Pro agility shuttle: 4.57-m sprint, COD of 180°, 9.14-m sprint, COD of 180°, 4.57-m sprint to the finish	Construct	Starters better than non-starters (1.0%, ES (d) = 0.29)	8 (10)
					60-yard shuttle: 4.57-m sprint, COD of 180°, 4.57-m sprint, COD of 180°, 9.14-m sprint, COD of 180°, 9.14-m sprint, COD of 180°, 13.72-m sprint, COD of 180°, 13.72-m sprint to the finish	Construct	Starters better than non-starters (0.4%, ES (d) = 0.17)	8 (10)
Huijgen et al. [105]	113	male	17.1 ± 0.7	Selected and deselected players of talent development programmes of professional soccer clubs (Netherlands)	Slalom Sprint: 30-m slalom section with 12 cones placed in a zig-zag pattern (horizontal and lateral displacement: 2 m)	Construct	Selected better than deselected (1.9%; ES (d) = 0.40)	7 (10)
					Slalom Dribbling Slalom sprint while dribbling a ball	Construct	Selected better than deselected (2.6%; ES (d) = 0.37)	7 (10)
Rebelo et al. [43]	180	male	18.2 ± 0.6	1st division elite, regional division non-elite (Portugal)	T Test (40 m): Linear sprinting (10 m), COD of 90° to the left (5 m), COD of 180° (10 m), COD of 180° to the left (5 m), COD of 90° to the left (10 m)	Construct	Elite better than non-elite (3.2%; ES (d): 0.90)	7 (10)
					Slalom Dribbling (approx. 32 m): 9 cones, each cone 2 m apart, slalom dribbling around the cones, COD of 180° and slalom dribbling back to the start	Construct	Elite better than non-elite (3.6%; ES (d): 0.42)	7 (10)
Kutlu et al. [106]	70	male	21.2 ± 3.0	Professional, amateur (Turkey)	T Test: Several CODs, forward sprinting, left- and right-side shuffling, and back pedaling; no further information on test procedures given	Construct	Professional faster than amateur (5.27%, ES (d) = 1.5)	5 (10)
					T Test with Ball: No information on test procedures given	Construct	Professional faster than amateur (3.92%; ES (d) = 0.81)	4 (10)
					Zig-Zag: No information on test procedures given	Construct	Professional faster than amateur (9.11%; ES (d) = 1.64)	4 (10)
					Illinois test: Accelerating, decelerating, several CODs of different angles; no further information on test procedures given	Construct	Professional faster than amateur (1.81%; ES (d) = 0.5)	5 (10)
Russel et al. [107]	20	male	19 ± 4	Recreational university reserve team, Professional Championship team (England)	Slalom Dribbling (approx. 20 m): 7 cones, each cone 3 m apart, 1st cone 1 m from start, slalom dribbling around the cones, finish 1 m from 7th cone	Construct	Professional better than recreational (2.4%; ES (d) = 0.37)	7 (10)
Keiner et al. [108]	111	male	U19, U21, Senior (not specified)	U19 and U21 of elite club, professional players of 1st and 2nd German division (Germany)	Equilateral triangle, 5 m side length 10-m sprint, 2 CODs of 60° after 2.5 m and 7.5m, 2.5-m sprint to the finish, split at 5 m	Construct	5 m: U21 (STG) better than U21 (CG) (3.1–3.4%, ES (d) = 1.0–1.4), U19 (STG) (3.4–4.1%, ES (d) = 0.85–0.94), professional (3.5–5.4%, ES (d) = 1.16–1.89), and U19 (CG) (5.6–7.0%, ES (d) = 1.42–2.01) 10 m: U21 (STG) better than U21 (CG) (3.1–4.6%, ES (d) = 1.25–1.33), U19 (STG) (3.2–3.5%, ES (d) = 0.85–0.98), professional (3.8–4.3%, ES (d) = 1.34–1.50), and U19 (CG) (6.6–7.9%, ES (d) = 2.06–2.42)	5 (10)

MQ–Methodological quality, maximal possible score in parenthesis; ES–Effect size; COD–change of direction; STG–Strength training group; CG–Control group

Subject characteristics and test execution were clearly depicted in most of the studies. In addition, the majority of studies used appropriate statistical methods at least to some extent. Conversely, only a small amount of studies stated the competence of the raters or described methodological aspects in sufficient detail, with blinding of the raters being stated in none of the studies.

### Study characteristics and main findings

#### Linear-sprint tests

Linear-sprint tests were examined 67 times. The distances investigated ranged from 5 to 200 m. The most frequent studied distances were 10, 20, and 30 m. In terms of construct validity, the test results between the playing levels differed between -1.6 and 5% (ES = -0.33–1.3), whereas positive values indicate that the higher-level players performed better than the lower-level players. Negative values indicate the opposite. Regarding criterion validity, the highest correlation coefficient found between test results and match parameters was r = -0.73.

Intraday reliability ranged from 0.17 to 0.99 (ICC) and from 0.7 to 7.8% (CV), whereas interday reliability ranged from 0.77 to 0.98 (ICC) and from 0.5 to 10.9% (CV).

Study findings in relation to the validity and reliability of linear-sprint tests are illustrated in Tables 2–3.

#### Repeated-sprint tests

Repeated-sprint tests were examined 15 times. The investigated tests incorporated 3 to 15 repetitions over distances ranging from 15 to 40 m with active and passive recovery between approximately 15 s and 1 min. The most frequent utilized tests comprised of 6 x 20-m sprints with approximately 20–25 s of active recovery (n = 3) and 7 x 30-m sprints with approximately 20–30 s of active or passive recovery (n = 3).

In terms of construct validity, the test results between the playing levels ranged from 0.3 to 2.7% (ES = 0.14–0.9) for the fastest time, between 0.4 and 2.6% (ES = 0.1–0.88) for the average time, and between 2.3 and 10.3% (ES = 0.83–5.5) for the total time. Results for the percent decrement ranged from -22.9 to 14.5% (ES = -0.4–0.39). Positive values indicate that the higher-level players performed better than the lower-level players. Negative values indicate the opposite. Regarding criterion validity, the highest correlation coefficient found between test results and match parameters was r = -0.51.

Intraday reliability was ICC = 0.75 and CV = 0.8% for the total time. Interday reliability was ICC = 0.88 and CV = 5.0% for the fastest time as well as ICC = 0.90 and CV = 5.0% for the average time. Moreover, ICCs and CVs ranged from 0.91 to 0.99 and from 0.8 to 5.0% for the total time and from 0.11 to 0.14 and from 16.8 to 46.0% for the percent decrement, respectively.

Study findings in relation to the validity and reliability of repeated-sprint tests are illustrated in Tables 4–5.

#### Change-of-direction sprint tests

Change-of-direction sprint tests were examined 60 times. The investigated distances ranged from 10 to 60 m including 1 to 9 directional changes of 45° to 270°. The most frequent studied tests were the T Test (n = 10), 505 test (n = 4), and zig-zag tests in various modifications (n = 5).

In terms of construct validity, the test results between the playing levels differed between -5.4 and 12.2% (ES = -1.89–1.64). Positive values indicate that the higher-level players performed better than the lower-level players. Negative values indicate the opposite. Regarding criterion validity, the highest correlation coefficient found between test results and match parameters was r = -0.56.

Intraday reliability ranged from 0.37 to 0.99 (ICC) and from 1.1 to 13.0% (CV), whereas interday reliability ranged from 0.63 to 0.98 (ICC) and from 0.8 to 4.0% (CV).

Study findings in relation to the validity and reliability of change-of-direction sprint tests are illustrated in Tables 6–7.

**Table 7:** Change-of-direction sprint tests (reliability).

Study	Population				Short description	Type	Results	MQ
	N	Gender	Age	Playing level (Country)				
Gelen [49]	26	male	23.2 ± 3.2	Professionals from 3rd division (Turkey)	Slalom Dribble (10 m): 4 cones, each cone 2 m apart, slalom dribbling around the cones	Intraday	ICC = 0.87–0.91	7 (14)
Bendiksen et al. [109]	11	female	21.0 ± 4.5	Professionals from 2nd best league (Norway)	Shuttle Sprint (40 m) 40-sprint, COD of 180° after 20 m, test is part of a soccer match simulation	Interday	10 m: ICC = 0.91; CV = 2.3% 20 m: ICC = 0.91; CV = 2.9%	8 (14)
Currell et al. [110]	11	male	21.4 ± 1	Recreational (not specified)	Running through a series of markers as quickly as possible, test is part of a soccer match simulation; no further information on test procedures given	Interday	CV = 1.2%	7 (14)
					Ball dribbling: Each participant had to negotiate a course of five cones set out directly behind one another as quickly as possible, test is part of a soccer match simulation	Interday	CV = 2.2%	7 (14)
Rouissi et al. [50]	31	male	17.42 ± 0.55	Professionals from 1st division (Tunisia)	10-m sprint, COD of 45° after 5 m	Interday	ICC = 0.88–0.89; CV = 1.2–1.8%	9 (14)
						10-m sprint, COD of 90° after 5 m	Interday	ICC = 0.87–0.88; CV = 1.5%	9 (14)
						10-m sprint, COD of 135° after 5 m	Interday	ICC = 0.92; CV = 1.2–2.2%	9 (14)
						10-m sprint, COD of 180° after 5 m	Interday	ICC = 0.89–0.94; CV = 1.6–1.9%	9 (14)
Emmonds et al. [56]	10	female	25.4 ± 7.0	Professional from highest division (WSL1) (England)	505 test (20 m): COD of 180° after 15 m, time taken 10–20 m	Intraday	ICC = 0.99; CV = 2.2%	8 (14)
Mujika et al. [57]	20	male	18.3 ± 0.6	Professional juniors at national level (not specified)	15-m sprint: Linear sprinting (3 m), slalom section (3 m), clearing a hurdle (2 m), linear sprinting (7 m)	Intraday	ICC = 0.92	9 (14)
Loturco et al. [58] & Loturco et al. [59]	27	male	18.4 ± 1.2	Professional U20, São Paulo state elite championship (Brazil)	Zig-zag test: 20-m sprint, 3 CODs of 100° every 5 m	Intraday	ICC = 0.96; CV = 2.4%	8 (14) 9 (14)
Miller et al. [111]	16	male	19.6 ± 0.8	NCAA Division III national championship (USA)	T Test (36.6 m) with contact mat: 9.1 m linear sprinting, 4.6 m shuffling to the left, 9.1 m shuffling to the right, 4.6 m shuffling to the left, 9.1 m backpedaling	Intraday	ICC = 0.86	6 (14)
Boone et al. [60]	289	male	25.4 ± 4.9	Professionals from 1st division (Belgium)	Shuttle sprint (5 x 10 m): 50-m sprint, 5 CODs of 180° every 10 m	Intraday	ICC = 0.81	5 (14)
Castillo-Rodríguez et al. [112]	42	male	20.11 ± 3.68	Amateur (Spain)	10-m sprint, COD of 90° after 5 m	Intraday	ICC = 0.81–0.88	5 (14)
						10-m sprint, COD of 180° after 5 m	Intraday	ICC = 0.83	5 (14)
Caldwell & Peters [71]	13	male	24 ± 4.4	Semi-professionals from nationwide conference north league (England)	Illinois test: Start from a lying position; no further information on test procedures given	Interday	ICC = 0.78	7 (14)
Thomas et al. [80]	12	male	17.3 ± 0.4	Semi-professionals from soccer acamedy (United Kingdom)	505 test	Interday	ICC = 0.99	7 (14)
Rey et al. [82]	18	male	26.6 ± 3.7	Professional (Spain)	T Test (36.6 m): 9.1 m linear sprinting, 4.6 m shuffling to the left, 9.1 m shuffling to the right, 4.6 m shuffling to the left, 9.1 backpedaling	Interday	ICC = 0.91	5 (14)
Rebelo et al. [43]	180	male	18.2 ± 0.6	1st division elite, regional division non-elite (Portugal)	T Test (40 m): Linear sprinting (10 m), COD of 90° to the left (5 m), COD of 180° (10 m), COD of 180° to the left (5 m), COD of 90° to the left (10 m)	Intraday	ICC = 0.95	6 (14)
						Slalom Dribbling (approx. 32 m): 9 cones, each cone 2 m apart, slalom dribbling around the cones, COD of 180° and slalom dribbling back to the start	Intraday	ICC = 0.99	6 (14)
Russel et al. [107]	20	male	19 ± 4	Recreational university reserve team, Professional Championship team (England)	Slalom Dribbling (approx. 20 m): 7 cones, each cone 3 m apart, 1st cone 1 m from start, slalom dribbling around the cones, finish 1 m from 7th cone	Interday	Mean ball speed during dribbling: ICC = 0.78; r = 0.78; CV = 2.4%	9 (14)
Di Mascio et al. [113]	11	male	17 ± 1	Elite U18 EPL (England)	Arrowhead Agility Test: Cones in an arrowhead shape, cones to indicate the start and finish line; no further information on test procedures given	Interday	CV = 0.8%	7 (14)
Silva et al. [34]	8	male	25.7 ± 4.6	Portuguese championship (Portugal)	T Test (36.6 m): Linear sprinting (9.1 m), COD of 90° to the left (4.6 m), COD of 180° (9.1 m), COD of 180° to the left (4.6 m), COD of 270° to the left (9.1 m)	Not Specified	ICC: 0.75–0.85	6 (14)
Mirkov et al. [88]	20	male	20.4 ± 1.8	Professionals from 1st selection of a premier national league team (Serbia)	Shuttle sprint (10 × 5 m): 50-m sprint, 10 CODs of 180° every 5 m	Intraday	ICC = 0.94; CV = 1.2%	9 (14)
						Zig-zag test: 20-m sprint, 3 CODs of 100° every 5 m	Intraday	ICC = 0.84; CV = 2.5%	9 (14)
						Zig-zag test with ball	Intraday	ICC = 0.81; CV = 3.3%	9 (14)
Meylan et al. [62]	20	female	18.2 ± 0.7	Professionals from national team (Top 10 in the world)	20-m sprint, COD of 90° after 10 m, with timing lights and GPS	Intraday	Timing lights: ICC = 0.81–0.93; CV = 1.1–2.4% GPS: ICC = 0.37–0.77; CV = 3.7–13.0%	6 (14)
Yanci et al. [66]	39	male	22.9 ± 2.8	Professionals from 3rd division (Spain)	Modified T Test (20 m): 5 m linear sprinting, 2.5 m shuffling to the left, 5 m shuffling to the right, 2.5 m shuffling to the left, 5 m sprinting back to the start line	Intraday	CV = 2.3%	9 (14)
						505 test (10 m): COD of 180° after 5 m, 5 m sprinting back to the start	Intraday	CV = 3.3%	9 (14)
						20-yard test (18.3 m): 4.6-m sprint, COD of 180°, 9.1-m sprint, COD of 180°, 4.6-m sprint	Intraday	CV = 1.8%	9 (14)
Shalfawi et al. [78]	20	female	19.4 ± 4.4	Well-trained players from 2nd division (Norway)	Sprint 9–3–6–3–9 m with 180° turns (30 m): 4 CODs of 180° every 3–9 m, 9-m sprint to the finish	Interday	ICC = 0.63	8 (14)
Los Arcos et al. [21]	42	male	23.2 ± 2.4	Professionals from 2nd and 3rd division (Spain)	Modified T Test (20 m): 5 m linear sprinting, 2.5 m shuffling to the left, 5 m shuffling to the right, 2.5 m shuffling to the left, 5 m sprinting back to the start line	Intraday	ICC = 0.80	9 (14)
						505 test (10 m): COD of 180° after 5 m, 5 m sprinting back to the start	Intraday	ICC = 0.87	9 (14)
						20-yard test (18.3 m): 4.6-m sprint, COD of 180°, 9.1-m sprint, COD of 180°, 4.6-m sprint	Intraday	ICC = 0.72	9 (14)
Pojskic et al. [114]	20	male	17.0 ± 0.9	Professionals at highest level of competition at their age (Sweden)	Soccer-specific test of change-of-direction speed (5 x 8 m): Sprinting to one of 4 cones, rebounding a ball in front of the cone, and returning to the start, 5 sprints per trial	Intraday	ICC = 0.92; CV = 5.9%	8 (14)
Kutlu et al. [90]	34	female	20.8 ± 1.9	Amateurs from university soccer team (Turkey)	Change-of-Direction and Acceleration Test (24 m): Linear sprinting (5 m), COD of 45°, linear sprinting (3 m), COD of 90°, linear sprinting (3 m), COD 90°, linear sprinting (3 m), COD of 45°, linear sprinting (10 m)	Interday	ICC = 0.98; CV = 4.0%	9 (14)
						Illinois test (approx. 60 m): Start from a standing position, linear sprinting (10 m), COD of approx. 180°, linear sprinting (10 m), COD of approx. 180°, slalom section (approx. 10 m), COD of 180°, slalom section (approx. 10 m), COD of approx. 180°, linear sprinting (10 m), COD of 180°, linear sprinting (10 m)	Interday	ICC = 0.98; CV = 4.0%	9 (14)
						T Test: No information on test procedures given	Interday	ICC = 0.95; CV = 4.0%	8 (14)
Silva et al. [85]	18	male	25.7 ± 4.6	Professionals from Portuguese elite championship (Portugal)	T Test (36.6 m): 9.1 m linear sprinting, 4.6 m shuffling to the left, 9.1 m shuffling to the right, 4.6 m shuffling to the left, 9.1 backpedaling	Not specified	ICC = 0.70–0.85	6 (14)
Sporis et al. [115]	150	male	19.1 ± 0.6	Professionals from 1st junior league (Croatia)	T Test (36.6 m): 9.1 m linear sprinting, 4.6 m shuffling to the left, 9.1 m shuffling to the right, 4.6 m shuffling to the left, 9.1 backpedaling	Intraday	ICC = 0.93; CV = 3.3%	11 (14)
						Slalom Test (approx. 22 m): 6 cones, each cone 2 m apart, 1st cone 1 m from start, slalom sprinting around the cones, COD of 180° and slalom sprinting back to the start	Intraday	ICC = 0.99; CV = 2.9%	11 (14)
						Sprint 4 x 5 m (20 m): 3 CODs of 90° or 180° every 5 m, 2-m sprint to the finish	Intraday	ICC = 0.98; CV = 4.3%	11 (14)
						Sprint with 90° turns (21 m): 6 CODs of 90° every 2–5 m, 5-m sprint to the finish	Intraday	ICC = 0.98; CV = 2.9%	11 (14)
						Sprint 9–3–6–3–9 m with 180° turns (30 m): 4 CODs of 180° every 3–9 m, 9-m sprint to the finish	Intraday	ICC = 0.95; CV = 5.1%	11 (14)
						Sprint 9–3–6–3–9 m with backward and forward running	Intraday	ICC = 0.95; CV = 5.6%	11 (14)

MQ–Methodological quality, maximal possible score in parenthesis; ES–Effect size; COD–change of direction, ICC–Intraclass correlation coefficient; CV–Coefficient of variation; GPS–Global positioning system; EPL–English premier league

#### Agility tests

Agility tests were examined 4 times. The investigated distances ranged from 8 to 40 m with 1 to 9 directional changes of 45° to 180°. Flashing light, video, and human stimuli were applied to indicate the directional changes. Each test was investigated once.

There were no studies investigating the construct or criterion validity of agility tests. Intraday reliability ranged from 0.70 to 0.88 (ICC) and from 3.7 to 4.9% (CV), whereas interday reliability was 0.70 (ICC) and ranged from 0.8 to 2.3% (CV).

Study findings in relation to the reliability of agility tests are illustrated in Table 8.

**Table 8:** Agility tests (reliability).

Study	Population				Short description	Type	Results	MQ
	N	Gender	Age	Playing level (Country)				
Bullock et al. [86]	18	male	18 ± 3	High-level amateurs from local soccer clubs (not specified)	Reactive Agility Test (approx. 9.4 m): Reacting to a video of a life-size soccer player dribbling the ball towards the player by sprinting in the same direction as the video; test is a part of a complex test	Interday	ICC = 0.70; r = 0.71; CV = 2.3%	8 (14)
Zois et al. [55]	10	male	23.3 ± 2.5	Amateurs from Serie D (Italy)	Reactive Agility Test (approx. 8 m): Reacting to a tester displaying different movements by sprinting in the same direction as the tester	Interday	CV = 0.8%	8 (14)
Pojskic et al. [114]	20	male	17.0 ± 0.9	Professionals at highest level of competition at their age (Sweden)	Soccer-specific test of reactive agility (5 x 8 m): Reacting to one of 4 LEDs on a cone by sprinting to and rebounding a ball in front of the cone, and returning to the start, 5 sprints per trial	Intraday	Protocol 1: ICC = 0.70; CV = 3.7% Protocol 2: ICC = 0.88; CV = 4.7% Protocol 3: ICC = 0.87; CV = 4.9%	10 (14)
McGawley & Andersson [83]	18	Male	23.0 ± 4.0	Semi- and fully-professional from 1st division (Sweden)	Modified T Test (40 m): Linear sprinting (10 m), random COD to the left or the right–example left side–COD of 90° to the left (5 m), COD of 180° (10 m), COD of 180° to the left (5 m), COD of 90° to the left (10 m)	Interday	CV = 0.8%	7 (14)

MQ–Methodological quality, maximal possible score in parenthesis; ES–Effect size

*–Selected parameters

%Dec–Percent decrement; FT–Fastest time; AT–Average time; TT–Total time, ICC–Intraclass correlation coefficient, r–Pearson’s r; CV–Coefficient of variation

#### Combinations

Combinations of the other test categories were examined 21 times. The investigated tests ranged from 3 to 10 repetitions over distances from 20 to 40 m with 1 to 5 directional changes of 45° to 180°. Both active and passive recovery ranging from approximately 15 to 40 s were utilized. Light stimuli were applied in all tests. The most frequent studied tests were the Bangsbo sprint test and the repeated shuttle-sprint test.

In terms of construct validity, the test results between the playing levels differed between 0.6 and 2.4% (ES = 0.44–0.82) for the fastest time, between 0.4 and 15.4% (ES = 0.28–15.24) for the average time, and between 0.4 and 9.7% (ES = 0.16–0.60) for the total time. Results for the percent decrement ranged from -23.4 to 45.9% (ES = -0.74–1.60). Positive values indicate that the higher-level players performed better than the lower-level players. Negative values indicate the opposite. Regarding criterion validity, the highest correlation coefficient found between test results and match parameters was r = -0.74.

Intraday reliability was ICC = 0.89 for the fastest time. Interday reliability ranged from 0.15 to 0.79 (ICC) and from 1.1 to 9.0% (CV) for the fastest time as well as from 0.58 to 0.81 (ICC) and from 0.9 to 10.0% (CV) for the average time. Moreover, ICCs and CVs ranged from 0.89 to 0.94 and from 0.8 to 10.0% for the total time, and from 0.17 to 0.49 and from 29.8 to 51.0% for the percent decrement, respectively.

Study findings in relation to the validity and reliability of combinations are illustrated in Tables 9–10.

**Table 9:** Combinations (validity).

Study	Population				Short description	Type	Results	MQ
	N	Gender	Age	Playing level (Country)				
Rampinini et al. [116]	18	male	26.2 ± 4.5	1st national league (one of the most important European leagues)	Repeated Shuttle-Sprint Test: 6 x 40-m sprints, COD of 180° after 20 m, 20 s passive recovery	Criterion	Test: FT, AT, %Dec Match: sprinting distance, very high-speed running distance AT and sprinting distance: r = -0.65 AT and very high-speed running distance: r = -0.60 No further significant correlations between FT and %Dec, and match parameters*	9 (14)
Di Mascio et al. [113]	48	male	Elite U18 EPL: 17 ± 1 Elite U18 EFL: 17 ± 1 Sub-elite U18: 17 ± 1	Elite U18 EPL, elite U18 EFL, sub-elite U18 (England)	Soccer-specific reactive repeated-sprint test: 8 x 30-m sprints, 3 random CODs to the left or the right of 45°, 135°, and 180°, 2 curved sprints, 30 s active recovery	Criterion	Test: TT Match: High-speed running (most intense 5-min period, whole match), total distance High-speed running most intense 5-min period: r = -0.55 –-0.74 High-speed running whole match: r = -0.55 –-0.67 Total distance: r = -0.25 –-0.66*	12 (14)
	106	Female, male	Elite U18 EPL: 17 ± 1 Elite U18 EFL: 17 ± 1 sub-elite U19: 18 ± 1 sub-elite U18: 17 ± 1 Elite senior female: 21 ± 3	Elite U18 EPL, Elite U18 EFL, sub-elite U19, sub-elite U18, Elite senior female (England)		Construct	TT: Elite U18 EPL better than sub-elite U19 (4.8%), sub-elite U18 (5.9%), and elite senior female (9.7%) Elite U18 EFL better than sub-elite U19 (3.5%), sub-elite U18 (4.6%), and elite senior female (8.3%)**	7 (10)
Huijgen et al. [105]	113	male	17.1 ± 0.7	Selected and deselected players of talent development programmes of professional soccer clubs (Netherlands)	Repeated Shuttle Sprint: 3 x 30-m sprints, starting every 20 s, 3 CODs of 180° after 5 m, 10 m, and 20 m	Construct	Selected always better than deselected FT: 1.7%; ES (d) = 0.55 TT: 1.4%; ES (d) = 0.47	7 (10)
					Repeated Shuttle Dribbling: Repeated Shuttle Sprint while dribbling with a ball	Construct	Selected better than deselected FT: 2.4%; ES (d) = 0.66 TT: 2.9%; ES (d) = 0.60	7 (10)
Dellal & Wong [96]	49	male	Senior, U19 (not specified)	Second team of a professional club, U19 (France)	Repeated change-of-direction test: 10 x 20-m sprints, 4 CODs of 100° every 4 m, 25 s active recovery	Construct	FT: Senior better than U19 (0.6%; ES (d) = 0.72) AT: Senior better than U19 (0.4%; ES (d) = 0.41) TT: Senior better than U19 (0.4%; ES (d) = 0.43) %Dec: U19 better than Senior (23.4%; ES (d) = 0.74)	5 (10)
Rampinini et al. [117]	23	male	professional: 25 ± 4 amateur: 26 ± 6	Professional from 3rd division, amateur from 6th division (not specified)	Repeated Shuttle-Sprint Test: 6 x 40-m sprints, COD of 180° after 20 m, 20 s passive recovery	Construct	Professional always better than amateur FT: 1.6%; ES (d) = 0.82 AT: 3.2%; ES (d) = 1.72 %Dec: 25%; ES (d) = 0.83	7 (10)
Wong et al. [97]	34	male	23.3 ± 3.6	Professional, college (not specified)	Repeated Change-of-Direction Test: 6 x 20-m sprints, 4 CODs of 100° every 4 m, 25 s active recovery	Construct	FT: Professional faster than college (1.8%; ES (d) = 0.47) AT: Professional faster than college (1.8%; ES (d) = 0.46) TT: Professional faster than college (1.8%; ES (d) = 0.48) %Dec: College faster than professional (0.8%; ES (d) = 0.02)	5 (10)
Impellizzeri et al. [118]	108	male	24 ± 4	Top-professional, mid-professional, amateur (not specified)	Repeated-shuttle-sprint ability test: 6 x 40-m sprints, COD of 180° after 20 m, 20 s passive recovery	Construct	FT: Top-professional better than mid-professional (1.1%; ES (d) = 0.44) and amateur (5.7%; ES (d) = 2.03) AT: Top-professional better than mid-professional (0.7%; ES (d) = 0.28) and amateur (2.8%; ES (d) = 0.96) %Dec: Top-professional better than mid-professional (35.3%; ES (d) = 1.08) and amateur (45.9%; ES (d) = 1.60)	8 (10)
Abrantes et al. [119]	146	male	1st national: 26 ± 3 2nd national: 24 ± 2 1st regional: 29 ± 5	1st national level professional, 2nd national level professional, 1st regional level semi-professional (Portugal)	Bangsbo sprint test: 7 x 34.2-m sprints, 3 CODs of 45° after 10 m, 90° after 17.1 m, and 45° after 24.2 m, 10-m sprint to the finish, 25 s active recovery	Construct	AT: 1st national better than 2nd national (6.1%; ES (d) = 4.37) and 1st regional (15.4%; ES (d) = 15.24)	6 (10)

MQ–Methodological quality, maximal possible score in parenthesis; ES–Effect size

*–Selected parameters

**–Data required to calculate ES not available

%Dec–Percent decrement; FT–Fastest time; AT–Average time; TT–Total time; COD–change of direction; EPL–English premier league; EFL–English football league

**Table 10:** Combinations (reliability).

Study	Population				Short description	Type	Results	MQ
	N	Gender	Age	Playing level (Country)				
Kaplan [120]	85	male	20.95 ± 3.8	Different amateur clubs (Turkey)	Bangsbo sprint test: 7 x 34.2-m sprints, 3 CODs of 45° after 10 m, 90° after 17.1 m, and 45° after 24.2 m, 10-m sprint to the finish, 25 s active recovery	Not Specified	TT: ICC = 0.94	5 (14)
Wong et al. (2012)[97]	34	male	23.3 ± 3.6	Professional, college (not specified)	Repeated Change-of-Direction Test: 6 x 20-m sprints, 4 CODs of 100° every 4 m, 25 s active recovery	Interday	FT: ICC = 0.79; CV = 9.0% AT: ICC = 0.80; CV = 10.0% TT: ICC = 0.80; CV = 10.0% %Dec: ICC = 0.17; CV = 51.0%	7 (14)
Impellizzeri et al. [118]	22	male	22 ± 1	Professional (not specified)	Repeated shuttle-sprint test: 6 x 40-m sprints, COD of 180° after 20 m, 20 s passive recovery	Interday	Short-term reliability (2–7 days) FT: ICC = 0.15 AT: ICC = 0.81 %Dec: ICC = 0.17	11 (14)
	30	male	25 ± 5	Professionals from national league (not specified)	see above	Interday	Long-term reliability (3 months) FT: ICC = 0.63, CV = 1.2% AT: ICC = 0.58; CV = 0.9% %Dec: ICC = 0.49; CV = 29.8%	10 (14)
Wragg et al. [121]	7	male	23 ± 4	National level student players (United Kingdom)	Modified Bangsbo sprint test: 7 x 34.2-m sprints, 3 random CODs to the left or to the right of 45° after 10 m, 90° after 17.1 m, and 45° after 24.2 m, 10-m sprint to the finish, 25 s active recovery	Interday	CV = 1.8%	9 (14)
Di Mascio et al. [113]	14	male	18 ± 1	Sub-Elite U19 (England)	Soccer-specific reactive repeated-sprint test: 8 x 30-m sprints, 3 random CODs to the left or the right of 45°, 135°, and 180°, 2 curved sprints, 30 s active recovery	Interday	TT: CV = 0.8% FT: CV = 1.1%	9 (14)
Brahim et al. [122]	27	male	17.6 ± 0.5	National team U19 (Norway)	Bangsbo sprint test: 7 x 34.2-m sprints, 3 CODs of 45° after 10 m, 90° after 17.1 m, and 45° after 24.2 m, 10-m sprint to the finish, 25 s active recovery	Interday	TT: ICC = 0.93	6 (14)
					12 x 20-m sprints, 3 CODs in a zig-zag pattern after 4.3 m, 12.5 m, and 15.7 m, 4.3-m sprint to the finish, 40 s active recovery	Interday	TT: ICC = 0.93	6 (14)
					Repeated shuttle-sprint test: 6 x 40-m sprints, COD of 180° after 20 m, 20 s passive recovery	Interday	TT: ICC = 0.89	6 (14)
Ruscello et al. [104]	17	male	21.9 ± 3.6	Professionals from Italian lega pro (Italy)	Shuttle sprint (2 x 15 m): 7 x 30-m sprints, COD of 180° after 15 m, exercise to rest ratio 1:5, passive recovery	Intraday	TT: ICC = 0.89	6 (14)
					Zig-zag: 7 x 30-m sprints, 5 CODs of 120° every 5 m, exercise to rest ratio 1:5, passive recovery	Intraday	TT: ICC = 0.89	6 (14)

MQ–Methodological quality, maximal possible score in parenthesis; ES–Effect size; %Dec–Percent decrement; FT–Fastest time; AT–Average time; TT–Total time; COD–change of direction

## Discussion

### Overview

This review examined the validity and reliability of different speed tests used in soccer, categorized into linear-sprint tests, repeated-sprint tests, change-of-direction sprint tests, agility tests, and combinations of these tests. In general, the high number of total studies and single tests included in this review highlights the importance of speed and speed testing in soccer. The majority of studies examined male players, which corresponds to the gender distribution of soccer players [123]. The tests were applied in a variety of performance levels, thereby allowing for both general and playing-level specific considerations.

Several different tests were identified in each category, while no accepted gold-standard tests seem to exist. The most studied tests were classified as linear-sprint tests and change-of-direction sprint tests, followed by combinations and repeated-sprint tests. Agility tests were the least studied. The amounts of tests in each category might be explained by differences relating to the complexity of the measurement set-up, test execution, and data analysis. For example, a 30-m linear sprint is relatively easy to conduct, while agility tests require the application of a stimulus which must be achieved through specific timing equipment incorporating flashing lights, life-size video clips or experienced humans [5,8].

Regardless of the test category, construct validity was investigated more frequently than criterion validity. This may be due to the additional match data needed for the same players in the latter case. Conversely, intraday and interday reliability were studied equally, although these approaches differ markedly in their organizational effort. However, in order to get a more holistic insight into the measurement properties of the tests, both types of validity and reliability should be assessed.

In the following paragraphs, the tests in each of the categories are discussed in relation to their validity and reliability. Based on this, recommendations for test selection in each category are given.

### Study characteristics and main findings

#### Linear-sprint tests

In terms of construct validity, the majority of studies report faster sprint times in favor of the higher-level players compared to the lower-level players. Such results have been found for both the comparison within professional players, e.g., national team vs. 1^st^ division players (trivial to small ES) [44,45], and the comparison between professional and amateur players (trivial to large ES) [36,37,40,43]. In addition, drafted players in try outs of a professional women’s soccer league demonstrated faster sprint times than non-drafted players (small to moderate ES) [42]. In line with this, starters outperformed non-starters of the same team (trivial to moderate ES), with a tendency to larger ES over longer distances [38,39,41].

However, tendencies for larger performance differences with increasing sprinting distance were not evident when all abovementioned studies were taken into consideration. Therefore, it might be concluded that all distances investigated (from 5 to 40 m) seem to be equally important in soccer, even though short sprints and accelerations (e.g., 10 m) occur more frequently than longer sprints (e.g., 40 m) during matches [2,3,124].

Some investigations reported faster sprint times for the players assigned to the lower playing level compared to those of the higher playing levels [35,47,48]. Besides the only trivial to small ES, in two studies, this finding was only obtained for a 10-m distance [48] and for males [47] with contrary results being obtained for a 20-m distance and females, respectively. Furthermore, in the third study [35], the lower-level players consisted of young elite amateur players who were training every day. Thus, both groups of players were considered as “high-level” players by the authors of that study.

In terms of criterion validity, only two studies were identified. Djaoui et al. [35] found a large relationship between the results of a 40-m sprint test and the maximal sprinting speed during matches. In addition, moderate to large relationships were reported for 5-m and 30-m sprints on the one side and high-intensity and sprinting distances during several periods of matches on the other side [34].

Considering both intraday and interday reliability, 40 studies report ICCs > 0.75 and CVs < 3.0% [21, 34, 43, 48–84]. The studies obtaining lower reliability (ICC ≥ 0.55 and CV ≤ 10.9%) integrated linear-sprint testing into complex tests [86] or match-simulation protocols [73,87] or required the players to adopt a defined running velocity at the start line [92]. In addition, it seems that the reliability decreases when considering longer terms, such as 6–12 months between measurements, with Pearson’s r and CV being 0.77–0.90 and 1.8–3.3%, respectively [44].

While more consistent reliability indices were obtained whilst utilizing established timing technologies such as timing lights and radar guns, varying results have been obtained for global positioning systems (ICC = 0.17–0.86; CV = 2.1–7.8%) [62,94]. Although not consistent over all studies, both intraday and interday reliability have been reported to be higher with increasing sprinting distance [21,45,66,67,80,88].

Given the results of the abovementioned studies, linear-sprint tests over distances up to 40 m possess acceptable construct validity and high intraday and interday reliability to assess linear-sprinting skills in soccer players.

#### Repeated-sprint tests

The identified repeated-sprint tests differ in their number of repetitions (3 to 15), the distance per repetition (15 to 40 m), and the type (active and passive) and duration (approximately 15 s to 1 min) of recovery per repetition. Common parameters derived from such tests include the fastest time, average time, total time, and percent decrement. The initial sprint time was reported less frequently.

The construct validity of repeated-sprint tests has been investigated in few studies (n = 5). In the majority of the studies, the higher-level players outperformed the lower-level players for all abovementioned parameters when comparing professional vs. semi-professional, college, university or regional level players; however, with considerably varying ES (trivial to very large) [96–99]. Only one study [100] found the lower-level players outperforming the higher-level players. However, this was true for percent decrement only. This result might be related to the low reliability of this parameter, which will be discussed later. Except for percent decrement, no parameter was superior to another in its ability to distinguish between playing levels. Interestingly, the largest ES between higher- and lower-level players were reported in a study with females [98]. This finding mirrors the observation that repeated-sprint bouts occur more frequent during matches of professional females in comparison with those of professional males [98,125,126].

Only one study examined the criterion validity of a repeated-sprint test (6 x 6-s sprints, 20 s passive recovery) in professional male players. A large correlation was found between percent decrement in the test and the frequency of high-intensity actions interspersed by recovery times ≤ 20 s during matches. In addition, a moderate correlation was reported between average velocity in the test and recovery time between high-intensity actions during matches [95]. Given the lack of further notable relationships between the test parameters and the frequency of repeated high-intensity bouts during matches, the authors question the criterion validity of this and similar tests. Indeed, more investigations using a similar study design are needed to confidentially draw conclusions with respect to criterion validity.

As a repeated-sprint test elicits considerable degrees of fatigue, multiple testing on one occasion (intraday reliability) appears to be rather inappropriate. Therefore, most of the studies reported interday reliability values (n = 6). Intraday reliability was examined less often (n = 2) and one study did not state the reliability type. ICCs for the average and total time exceeded 0.75 in all studies and were mostly higher than 0.90 while CVs were lower than 3.0% in 7 out of 9 studies [78, 81, 83, 97, 98, 101–103]. The reliability of the fastest time was 0.88 and 5.0% for ICC and CV, respectively [97]. Conversely, the percent decrement as a measure of fatigue was markedly less reliable (ICC ≥ 0.11, CV ≤ 46.0%) [81,98]. Pacing strategies of the players throughout the sprints was stated as a possible reason [127].

No differences between different recovery durations and modes were obvious regarding validity and reliability. However, the recovery duration should be short enough (e.g., < 30 s) to provoke the occurrence of fatigue [13]. Additionally, the recovery mode should be active in order to replicate the match demands [95].

The use of repeated-sprint tests has been criticized by some authors [2,128]. Their criticism is based on the very large correlations between the fastest time, average time and total time of such tests on the one side and results of single linear-sprint tests on the other side. Additionally, the low reliability of fatigue measures such as the percent decrement questions the additional benefits derived from repeated-sprint tests compared to linear-sprint tests. Nevertheless, based on the studies included in this review, repeated-sprint tests differing in the number of repetitions, the distance per repetition, and the recovery phases possess acceptable levels of construct validity and high levels of reliability for examining repeated-sprinting skills in adult soccer players regarding all parameters, except for percent decrement.

#### Change-of-direction sprint tests

A plethora of change-of-direction sprint tests has been developed and introduced into soccer. Some of these tests carry the word "agility" in their name (e.g., “Illinois agility run”, “Agility T Test”) but do not contain a response to a stimulus. Therefore, they were classified as change-of-direction sprint tests in this review. Change-of-direction sprint tests vary in their total distance (10–60 m) as well as number (1–9) and angles (45–270°) of directional changes. A frequently applied type of test involves shuttle sprints, where players sprint to a line, change the direction by 180°, and sprint back. Furthermore, test set-ups using zig-zag or slalom patterns are common. In addition, some popular tests were originally developed for sports other than soccer, such as the 505 test, Illinois test, and T Test.

The construct validity of change-of-direction sprint tests has been evaluated in a number of investigations (n = 14). As with linear-sprint tests and repeated-sprint tests, the higher-level players obtained faster times than the lower-level players in the vast majority of studies (n = 13). This applied to the comparison of starters vs. non-starters in a professional team (trivial to small ES) [41], professional vs. amateur players (small to large ES) [106], 1^st^ division vs. regional division players (moderate ES) [43], seniors vs. juniors of the same professional club (large ES) [47] and selected vs. deselected players in talent a program (small ES) [105]. Similar results were obtained when players were required to dribble a ball, commonly in a slalom or zig-zag manner (trivial to large ES) [43,47,105–107].

In contrast, the study of Keiner et al. [108] showed superior performance of U21-players of a professional soccer club compared to professional adult players. However, this was particularly evident for a group of U21-players who had performed a specific strength training program for the two proceeding years. In contrast, no detailed information was provided relating to the training contents of the professional adult players.

Only one study addressing the criterion validity of change-of-direction sprint tests met the inclusion criteria [34]. This study investigated the relationships between the results of the T Test and match parameters. Compared to 5-m and 30-m sprints, as depicted above, markedly lower relationships were evident. This finding particularly applied for the correlation between the T Test and sprinting distances during several periods of the match [34]. Therefore, it might be concluded that a high change-of-direction performance translates into sprinting behavior during matches only to a limited extent. Possibly, other match parameters that reflect change-of-direction behavior more directly might represent a more suitable alternative.

A considerable number of studies (n = 27, encompassing 45 tests) reported intraday or interday reliability of various change-of-direction sprint tests with ICCs usually exeeding 0.75 and CVs lower than 3.0% [34, 43, 49, 50, 56–60, 71, 80, 82, 106, 108, 109, 111]. Similar reliability was demonstrated in the four studies that included ball dribbling into the test [43,88,107,110].

Conversely, some studies report high relative reliability (ICCs 0.92–0.99) and somewhat lower absolute reliability (CVs 2.9–5.9%) [114,115]. Lower reliability was reported for shuttle sprints over 18.2 m (ICC = 0.72) [21] and 30 m (ICC = 0.63) [14].

As with linear-sprint testing, a change-of-direction sprint test using a global positioning system was reported less reliable (ICCs 0.37–0.77; CVs 3.7–13.0%) [62], supporting the utilization of appropriate timing technologies during speed testing [32].

The high number of change-of-direction sprint tests and the large differences in test design highlight the lack of an accepted gold standard [129]. However, some popular tests have been evaluated in several studies, such as the 505 test or the T Test. Several modifications of these tests have been applied. For example, the linear-sprint phase prior to the directional change of 180° in the 505 test varies between 5 m and 15 m in the literature [21,56,66,80]. Regarding the T Test, as many as six different types of this test have been used, differing in the total distance (20–40 m), the type of locomotion (shuffling, backpedaling, and sprinting), and the inclusion or exclusion of ball dribbling [21,34,43,66,82,85,90,106,111,115]. One study even added a visual stimulus prior to changing direction, leading this modification to be classified as an agility test [83]. Despite these modifications, all types of the 505 test and the T Test have been shown to be valid (T Test: ES = 0.62–1.50 in favor of the higher-level players) and/or reliable (505 test: ICC = 0.87–0.99, CV = 2.2–3.3%; T Test: ICC = 0.70–0.95, CV = 0.8–4.0%).

While many tests, including the 505 test and the T Test, do not mimic the match demands [2], the confirmed validity and reliability of these two tests for assessing change-of-direction sprinting skills through a number of studies allow their application until more game-specific tests are thoroughly evaluated.

#### Agility tests

Since the introduction of a classic agility test for invasion sports by Sheppard et al. [130], this test has been evaluated and modified for the specific demands of different sports, such as Australian football, basketball, netball or rugby [5,131].

With respect to the inclusion criteria of this review, no study was identified that evaluated the validity of an agility test in soccer players. This is somewhat surprising as agility tests have been shown to possess high levels of construct validity by discriminating between playing levels in Australian football and rugby league, while change-of-direction sprint tests did not [12]. This finding is mainly attributed to the superior anticipation and decision-making skills of higher-level players [5]. It should be noted that studies examining the construct validity of such tests in soccer exist. However, either the (sub-)sample investigated for this specific outcome was too young to be considered for this review [114] or the population also included sports other than soccer (e.g., futsal) [132]. Although more complex than capturing the number of sprints or maximum speed during matches, methods for analyzing decision-making during training and matches have already been applied to soccer and might serve as a foundation for evaluating the criterion validity of agility tests [133,134].

Conversely, the reliability of agility tests has been addressed in four studies, all of them relating to interday reliability [55,83,86,114]. Two of the tests used flashing lights as a stimulus (ICCs 0.70–0.87; CVs 0.8–4.9%) [83,114]. One study [55] adopted the classic agility test by Sheppard et al. [130], which requires the players to respond to different movements of a tester (human stimulus) by sprinting in the same direction as the tester (CV = 0.8%). The last study examined agility as a part of a complex test [86]. Here, players respond to a video of a life-size soccer player dribbling the ball towards the player by sprinting in the same direction as the video (ICC = 0.70; CV = 2.3%). The slightly lower reliability of agility tests compared to the other test categories might be attributed to the complexity of such tests, incorporating both physical and perceptual-cognitive aspects of speed. While several parameters can potentially be investigated during agility tests, such as the response time at the start, the decision-making time or the response accuracy [5], the abovementioned studies were limited to the total time to complete the test.

In terms of the applied stimuli, it has been shown in other sports (e.g., Australian rules football, field hockey) that humans or video sequences appear to be more appropriate than flashing lights when examining construct validity [5]. This seems reasonable as the latter does not allow higher-level players to utilize their anticipation and decision-making skills, but simply to react to a non-specific signal [135]. Given the small total number of investigations and the lack of studies using humans or video sequences as a stimulus, it can be concluded that the soccer-specific agility research is still in its infancy.

#### Combinations

This test category combines elements of two or more of the abovementioned test categories. Most of the studies examined pre-planned repeated change-of-direction sprint tests with or without ball dribbling (10 studies encompassing 12 tests), while two studies analyzed repeated change-of-direction sprint tests in response to a stimulus. Thereby, such tests comprise elements of repeated-sprint tests and change-of-direction sprint tests, and sometimes even those of agility tests. Similar to repeated-sprint tests, the fastest time, average time, total time, and percent decrement are commonly investigated during such tests. The most utilized tests were the (modified) Bangsbo sprint test [119–122] and the repeated shuttle-sprint test [116–118,122].

The construct validity of combination tests was supported in the vast majority of studies for most of the parameters in question. Specifically, the higher-level players performed better than their lower-level counterparts when comparing professional vs. semi-professional players (small to very large ES) [118,119], professional vs. amateur players (trivial to very large ES) [97,117,118], 2^nd^ team vs. U19 players of a professional club (small to moderate ES) [96] or selected vs. deselected players of a talent development program (small to moderate ES) [105]. Similarly to the results of the repeated-sprint tests, the percent decrement was not always able to discriminate between playing levels, with the lower-level players obtaining better scores in some studies (trivial to moderate ES) [96,97]. All other parameters were able to distinguish between playing levels.

The criterion validity of combination tests has been evaluated in two studies [113,116]. In the study of Rampinini et al. [116], the average time of a repeated shuttle-sprint test was largely correlated to the sprinting distance and very high-intensity running distance during professional matches. However, no notable relationships were evident between the fastest time or percent decrement and match variables. The second study analyzed a reactive repeated-sprint test involving changes of direction in response to a light stimulus [113]. The authors found large to very large correlations between the total time of the test and match parameters related to high-speed running. Small to large associations were reported for the total distance covered during matches [113].

In terms of reliability, the interday reliability of combination tests was addressed in a number of studies (5 studies encompassing 7 tests), while the intraday reliability was examined less frequent (1 study encompassing 2 tests). Varying results were obtained for different parameters. ICCs and CVs for the average time and total time were > 0.75 and < 2.0%, respectively, in most studies [104,113,118,120–122]. However, high CVs of 10.0% have also been found for these parameters [97]. Moreover, one study reported low relative reliability for the fastest time (ICC = 0.15) [118], while high absolute reliability (CV = 1.1%) was evident for the same parameter in another study [113]. More consistently, percent decrement was found to not be reliable (ICC = 0.17, CV = 51.0%) [97,118]. In addition, the relative reliability in long-term (3 months between occasions) seems to be somewhat lower compared to short-terms (ICC for average time 0.58), while the absolute reliability remains high (CV for average time 0.9%) [118].

In sum, the total and average time possess the highest degree of validity and reliability. Specifically, this was confirmed for the Bangsbo sprint test and the repeated shuttle-sprint test in a number of studies. Moreover, it should be noted that although evaluated in a single study only, the validity and reliability was confirmed for the reactive repeated-sprint test, which has been designed on the basis of match analysis.

#### Limitations

The findings of this systematic review should be interpreted in light of its limitations. We did not conduct an updated search that included studies published after May 2018. In addition, only studies examining soccer players with an average age of 17 years or above were considered. This automatically excludes investigations in younger age groups [9], which could have broadened the database. However, the number of included articles (n = 90) was already high in this review and results from other sports, although related, or differing age groups may not always be transferable [136].

We excluded investigations applying manual timing due to large absolute errors and issues relating to inter-rater reliability with this timing technology [32]. While this approach further reduces the available database, it ensures that an appropriate timing technology has been used in the studies, thereby accounting for adequate methodological quality in this regard.

The methodological quality of the construct and criterion validity studies was rated as high, while the scores of the intraday and interday reliability studies were somewhat lower. The latter finding might be explained by the inclusion criteria, as there was no restriction on the type of studies. Therefore, studies in which the reliability assessment was not the main aim were also included. While being well-designed for their primary aim (e.g., the evaluation of a training intervention), the necessary information for the reliability assessment were not always given.

In addition, the assessment of methodological quality itself should be viewed critically. Unfortunately, no assessment tool was applicable without modifications for the purpose of this review. In this context, another frequently used tool for the evaluation of measurement properties, the COSMIN checklist [137], seems more appropriate in relation to questionnaire-based studies [138] than for performance testing. Therefore, we made use of the critical appraisal tool by Brink & Louw [31] including some modifications, which promised a more suitable assessment of methodological quality of performance testing.

Another limitation might be position-specificity. We reported study results for all players of a team as a whole, thereby not accounting for position-specific demands which could lead to differing validity and reliability of speed tests and, therefore, specific test recommendations for each position [88,139].

### Further considerations and future research

Although a test may have shown to be valid and reliable, it does not automatically guarantee that the derived results provide new and useful information to the coach and the individual players [140]. While this issue seems still to be discussed [141], methodological barriers to data collection and analysis are overcome by modern technologies. As a result, researchers can better identify crucial factors of (speed) performance in soccer and consequently to develop tests with direct impact on coaches and players [140]. One solution might be the implementation of test designs based on detailed analysis of match demands. In fact, few studies clearly stated such an approach (e.g., [98,113]). However, this seems promising for future studies. Based on this, more studies are needed examining the relationship between test results and match parameters (criterion validity) throughout all test categories.

Besides intraday and interday reliability, it is of further interest to know if small performance changes can be identified using a specific test [142,143]. In particular, this becomes a matter at a professional level, where performance gains are usually small [144]. This test property, commonly referred to as usefulness, is determined through the ratio of the intra-individual variability and the so-called smallest worthwhile change (SWC) [143]. While the intra-individual variability is usually expressed as a CV, the SWC can either be calculated as 0.2 x standard deviation of a given population, representing a small effect, or a pre-defined threshold. Given the example of a 20-m linear-sprint test, Haugen et al. [2] stated that the SWC relates to approximately 0.02 s when expressed as a small effect. Considering a real-world scenario, a gap of 30 cm to 50 cm might be decisive in a sprint duel of two players. In this case, the SWC as a pre-defined threshold corresponds to 0.04–0.06 s over a 20-m distance. These approaches might not only be applied to linear-sprint testing, but also to the other test categories. However, being reported scarcely in the identified studies, the usefulness was not included in this review. Indeed, it has been highlighted that this test property is population-specific to great extends and, therefore, should be determined for each investigation or team separately [142].

Although demonstrating good validity and reliability, the value of repeated-sprint tests has been questioned, as mentioned above. As repeated accelerations have been found to occur much more frequently during matches [3], the concept of repeated-acceleration bouts has recently been introduced [125,145]. Therefore, the development and evaluation of repeated-acceleration tests should be subject of further investigations.

Lastly, agility tests are underrepresented compared to the other test categories. Based on the promising results from related sports evaluating such tests [5] and the increasing overall game speed [1], requiring the players to make fast decisions and perform an adequate motor response, more research with respect to agility tests is recommended. Particularly, tests using scenarios close to the game and specific stimuli seem appropriate.

## Conclusion

Speed is considered a crucial factor for overall performance in soccer. As most of the test categories evaluated in this review share a relatively low common variance, they represent rather independent skills. Therefore, no single test is appropriate to measure all aspects of speed concurrently, thus, a comprehensive examination of speed should cover all test categories.

Linear-sprint tests over various distances (5 to 40 m) can be used to determine acceleration and maximal speed. Thereby, such tests have been shown to be able to distinguish between playing levels, to correlate with sprint-related parameters during matches, and to possess high levels of reliability. Although criticized for not replicating the match demands, repeated-sprint tests of different number of repetitions, distances per repetition as well as types and durations of recovery have been reported to be valid in terms of discriminating playing levels and to be highly reliable. However, this specifically applies to the total time and the average time of such tests, while the use of percent decrement should be treated with caution. A high number of studies identified addressed change-of-direction sprint tests. Such tests vary dramatically in their total distance, number and angles of directional changes, and often do not mimic the match demands. Nevertheless, a number of tests, including the 505 test and T Test, possess high construct validity and reliability, thereby supporting their utilization in soccer. Conversely, agility tests have been investigated scarcely. While no information on the validity of agility tests is currently available, acceptable but slightly lower reliability compared to the other categories has been reported for tests applying flashing lights, video sequences, and humans as a stimulus. Combinations include elements of two or more test categories, commonly those of repeated-sprint and change-of-direction sprint tests and sometimes even agility tests. The total and average time possess the highest degree of validity and reliability, most frequently reported for the Bangsbo sprint test and the repeated shuttle-sprint test.

As currently stated, there is a lack of an accepted gold standard test in most of the categories. Researchers and practitioners might base their test selection on the comprehensive validity and reliability database provided in this review.

## Supporting information

S1 Table PRISMA checklist. (DOC)

S1 Text Full electronic search strategy for PubMed. (DOCX)
