# Supplementary material for: Validity and reliability of speed tests used in soccer: A systematic review
Source: PLoS One. 2019 Aug 14;14(8):e0220982. doi: 10.1371/journal.pone.0220982 (PMC6693781; doi:10.1371/journal.pone.0220982)
Supplement: S1 Text — (DOCX) [file pone.0220982.s002.docx]

**S1 Text. Full electronic search strategy for PubMed.**

(((soccer OR football)) AND (psychometric OR measurement OR reliability OR repeatability OR reproducibility OR measurement error OR consistency OR smallest worthwhile change OR minimal detectable change OR typical error OR usefulness OR validity OR logical OR construct OR convergent OR discrimination OR match performance OR gold standard OR level OR standard)) AND (speed OR quickness OR sprint OR acceleration OR maximum speed OR linear OR change of direction OR repeated sprint ability OR agility OR reactive agility OR physical OR unplanned OR unanticipated OR test OR testing)
